# Prevalence, trajectories, and determinants of television viewing time in an ethnically diverse sample of young children from the UK

**DOI:** 10.1186/s12966-017-0541-8

**Published:** 2017-07-06

**Authors:** Sally E. Barber, Brian Kelly, Paul J. Collings, Liana Nagy, Tracey Bywater, John Wright

**Affiliations:** 1 Bradford Institute for Health Research, Bradford Royal Infirmary, Duckworth Lane, Bradford, BD9 6RJ UK; 2 Faculty of Health Studies, University of Bradford, Richmond Rd, Bradford, BD7 1DP UK; 3 Department of Health Sciences, Faculty of Sciences, University of York, Area 2, Seebohm Rowntree Building, York, YO10 5DD UK

**Keywords:** Sedentary behaviour, Screen-time, Television, Early childhood, Ethnic minority, Deprivation, Prospective longitudinal

## Abstract

**Background:**

Excessive screen viewing in early childhood is associated with poor physical and psycho-social health and poor cognitive development. This study aimed to understand the prevalence, trajectory and determinants of television viewing time in early childhood to inform intervention development.

**Methods:**

In this prospective longitudinal study, mothers of 1558 children (589 white British, 757 Pakistani heritage, 212 other ethnicities) completed questionnaires when their children were approximately 6, 12, 18, 24 and 36 months old. Mothers answered questions about their own and their child’s TV-time. TV-time trajectories were estimated by linear longitudinal multilevel modeling, potential determinants were considered in models.

**Results:**

The modelled trajectory estimated that 75% of children aged 12 months exceeded guidelines of zero screen-time. At 12 months of age an accelerated increase in TV-time was observed (<1 h/day at 14 months, >2 h/day by 30 months old). For every hour of mothers’ TV-time and every hour the TV was on in the home, children’s TV-time was 8 min and 1 min higher respectively at 6 months old (*P* < 0.05), and 15 min and 3 min higher respectively at 36 months old (*P* < 0.05). Children whose mothers did not agree that it was important their child did not watch too much TV, had 17 min more TV-time than their counterparts (*P* < 0.05). Children of first time mothers had 6 min more TV-time (*P* < 0.05). At 12 months of age, children of mothers experiencing stress watched 8 min more TV (*P* < 0.05). By 36 months, children of Pakistani heritage mothers had 22 min more TV-time than those of white British mothers (*P* < 0.05), and an additional 35 min of TV-time if their mother was not born in the UK (*P* < 0.05).

**Conclusions:**

High levels of TV-time were prevalent. Intervention developers should consider targeting interventions before 12 months of age. Modifiable determinants included mothers’ own TV-time, the time the television is on in the home and mothers’ attitude towards child TV-time. These behaviours may be key components to address in interventions for parents. Mothers experiencing stress, first time mothers, and Pakistani heritage mothers (particularly those born outside of the UK), may be priority groups for intervention.

## Background

The amount of screen time (i.e. using a device such as a TV, computer, games console, mobile phone or tablet) that children engage in is associated with a number of adverse health and educational outcomes including; higher risk of obesity [1–4], poor metabolic profile [5], poor fitness later in life [6], poor cognitive development (e.g. poor short term memory skills, poor reading, language and mathematics development) [7] and adverse psychosocial health [8]. Currently Canada and Australia recommend no screen time exposure in children under 2 years old and less than an hour a day for 2–5 year olds [9, 10]. In the US it is recommended that digital media use (except video-chatting) is avoided in children younger than 18 to 24 months, screen time of children aged 2–5 year old should be limited to 1 h/day which is high quality, co-viewed with, and re-taught by parents [11].

A recent systematic review [12] reported that the proportion of children aged under two years old meeting zero screen time recommendations ranged from 2.3% in a study from Thailand to 83% in a study from the US. The review concluded that by two years of age the majority of children were already engaging in high levels of screen time and were exceeding recommendations [12].

Children from ethnic minority groups (including South Asian children) and children with low socioeconomic status (SES) have a greater risk of obesity than their Caucasian or high SES counterparts [13]. Young children from ethnic minority groups (specifically Black and Hispanic children in the US) are consistently reported to have greater screen exposure (i.e. the amount of time the child was in the room with a screen on) than Caucasian children [14]. Outside the US, differences in screen time between young ethnic minority and ethnic majority children have not been investigated. In the US and the UK, older children and adolescents with a lower SES are consistently reported to engage in higher levels of screen time [15–17]. However, the association between indicators of SES and screen time in early childhood is inconsistent between studies [14]. Given that TV-time is associated with a small but significant increase in BMI in early childhood [4], reducing TV-time for ethnic minority children, and children from low SES families, could be targeted by interventions to address the inequalities seen in obesity levels.

Screen viewing behaviour is relatively stable over time, and tracks moderately through childhood and adolescence [18]. However, in early childhood the behaviour may be less stable and more malleable, thus early intervention may be most beneficial [19]. For young children, watching TV is the screen behaviour that contributes the most to daily screen time [20, 21]. Ascertaining determinants of TV-time in young children will help to identify at risk groups for intervention and inform the design of behaviour change interventions to reduce TV-time during early childhood. A recent systematic review found associations between screen time (mostly TV-time) and the following modifiable variables in children aged up to 3 years old: Child’s BMI (4/4 studies), mother’s screen time (3/3 studies) and mother’s distress/depression (5/8 studies) [14]. Cognitive stimulation in the home environment was negatively associated with screen time (2/3 studies). However, none of these studies were conducted in the UK, or included children of South Asian ethnicity. The current study aimed to describe the prevalence, trajectories, and determinants of TV-time in an ethnically diverse (predominantly white British and Pakistani ethnicity) sample of children from 6 months through to 36 months old, living in the City of Bradford, West Yorkshire, UK.

## Methods

### Participants and setting

Born in Bradford (BiB) is a longitudinal multi-ethnic birth cohort study [22]. The study recruited pregnant women between 2007 and 2010 in Bradford, a city with high levels of socio-economic deprivation and ethnic diversity. Pregnant women were approached to take part whilst attending a routine hospital appointment at 26–28 weeks gestation. The full BiB cohort recruited 12,453 women during 13,776 pregnancies, at which time a baseline questionnaire was completed by interview with a trained study administrator. Women who were pregnant between August 2008 and March 2009 and who agreed to take part in the full cohort were also invited to take part in a sub-study named BiB1000 (*n* = 1735) at the same time. This study involved detailed follow-up appointments conducted in the home or clinic when children were aged approximately 6, 12, 18, 24, and 36 months [23]. A full description of the methods and data collected in BiB1000 is presented in the published protocol [23]. Informed consent was acquired prior to data collection and ethical approval for all aspects of the research was granted by Bradford Research Ethics Committee (Ref 07/H1302/112).

Of all children recruited to BiB1000, 1558 (90%) had at least one follow-up questionnaire completed; these children were included in the current study. Over half of the included children (*n* = 812) had questionnaires completed at all five follow-up time points, whereas 7% (*n* = 112) completed only one follow-up questionnaire. Four children died and 54 withdrew from the study, before the 36-month-old visit. Data for these children were excluded from the analysis. Eighty-two per cent of the sample completed the 6 and 12 and 18 month old visits; the children’s mean age (standard deviation) at these time points were 6.7 (0.74) months, 12.7 (0.99) and 18.7 (0.98) respectively. At the 24 months and 36 month old visits 79% completed questionnaires; the children’s mean age at these visits were 25.3 (0.95) months and 37.0 (0.85) months respectively. Data were collected between 2008 and 2013.

### TV-time, behaviours and attitudes

Information about child and mothers’ daily TV-time (time spent watching TV or DVDs) was collected at each follow-up visit (when children were 6, 12, 18, and 24, and 36 months old) using questions from the EPIC Norfolk questionnaire [24] which has been validated for use in adults [24]. At the 24-month visit, mothers were additionally asked to report how long (in hours and minutes) the television was on in the home, on an average week and weekend day; it was stressed that this included time when the TV was on but not being watched. They were asked whether they think it is important their child does not watch too much TV (response options were agree/not agree), and how often they limited their child’s TV/DVD viewing (questions were from the validated Southampton Women’s Survey Questionnaire [25], (response options collapsed to: never or rarely/sometimes or often).

### Socio-demographic variables

Mother’s self-defined ethnicity at baseline was used to define the ethnicity of her offspring according to the ethnic group classification system used in the 2001 UK Census [26]; children were categorised as white British, Pakistani or Other (a group that comprised all other ethnic groups that were too small to analyse separately). Mothers’ country of birth was collected from the baseline questionnaire (UK, Pakistan, Other) and mothers’ age at delivery, child sex, and parity, were obtained from the hospital maternity system. Mothers’ height and weight were recorded during pregnancy at registration in the maternity unit (around 10 weeks gestation) and extracted from hospital maternity systems; body mass index (BMI) was calculated. Child’s length and weight at the 24-month-old visit was measured and BMI z-scores were calculated.

Mothers’ SES was measured using a number of variables: self-reported financial situation, education level, housing tenure and neighbourhood material deprivation (using Index of Multiple Deprivation 2010; [27]). While it is the case that the participants were far more likely to be in a materially deprived neighbourhood (as shown in Table 1, only 1.5% were in the least deprived quintile score) the models used this deprivation score as a dichotomous variable. In the models the effect of being in the most deprived quintile (68%) was contrasted with not being in the most deprived quintile (32%), in order to maximise the variation – the most materially deprived neighbourhoods compared to the rest. Mothers’ non-specific psychological stress was measured using a validated 6-item questionnaire [28] administered at the 12- and 24-month-old visits. Mothers who scored four or more (out of a possible score of 24) were considered to be experiencing non-specific psychological stress [28]. This score has been found to discriminate between Diagnostic and Statistical Manual of Mental Disorders edition 4 (DSM-IV) cases and non-cases [28].

**Table 1:** Participant characteristics

	All	White British	Pakistani	Other
	*n* = 1558	*n* = 589	*n* = 757	*n* = 212
Mother				
Age at delivery: mean (Std. Dev.) (missing data, *n* = 15)	27.5 (5.74)	26.9 (6.11)	27.7 (5.15)	28.3 (5.78)
Country of Birth: n (%) (missing data, *n* = 0)				
UK	970 (62.3)	581 (98.6)	311 (41.1)	78 (36.8)
Pakistan	441 (28.3)	0	438 (57.9)	3 (1.4)
Other	147 (9.4)	8 (1.4)	8 (1.1)	131 (61.8)
BMI at registration (~10 weeks gestation): mean (Std. Dev.) (missing data, *n* = 60)	25.9 (5.74)	26.9 (6.18)	25.2 (5.41)	25.5 (5.22)
Neighbourhood material deprivation (IMD 2010): n (%) (missing data, *n* = 0)				
Quintile 1: Most deprived	1060 (68.0)	311 (52.8)	604 (79.8)	145 (68.4)
Quintile 2	286 (18.4)	133 (22.6)	111 (14.7)	42 (19.8)
Quintile 3	154 (9.9)	95 (16.1)	37 (4.9)	22 (10.4)
Quintile 4	34 (2.2)	30 (5.1)	2 (0.3)	2 (0.9)
Quintile 5: Least deprived	24 (1.5)	20 (3.4)	3 (0.4)	1 (0.5)
IMD Score: Mean (Std. Dev.)	42.9 (17.5)	37.2 (19.1)	46.7 (14.8)	45.2 (17.8)
Self-reported financial situation: n (%) (missing data, *n* = 8)				
Comfortable	409 (26.4)	137 (23.3)	209 (27.8)	63 (29.9)
All-right	648 (41.8)	254 (43.3)	304 (40.4)	90 (42.6)
Difficult	493 (31.8)	195 (33.3)	240 (31.9)	58 (27.5)
Mother non-specific psychological stress score when child was 12 months old (higher = greater stress; range 0–24) (missing data *n* = 285)	3.28 (3.89)	3.38 (3.90)	3.20 (3.89)	3.26 (3.82)
Percentage score 4 or more	35.6%	36.2%	35.1%	35.3%
Mother TV attitude when child was 24 months old: mother does not agree that it is important their child does not watch too much TV (missing data, *n* = 10)	23.3%	21.3%	25.2%	21.5%
Hours TV is on in house: Mean (Std. Dev.) (missing data, *n* = 364)	7.8 (4.0)	7.3 (3.9)	8.5 (4.0)	7.1 (4.1)
Whether mother restricts child TV viewing: n (%) (missing data, *n* = 377)				
Never	506 (42.8%)	147 (33.6%)	306 (51.8%)	53 (34.6%)
Occasionally	370 (31.3%)	145 (33.2%)	172 (29.1%)	53 (34.6%)
Everyday	305 (25.8%)	145 (33.2%)	113 (19.1%)	47 (30.7%)
No previous children (missing data, *n* = 35)	39.0%	47.3%	31.3%	43.5%
Child				
BMI 24 month: mean (Std. Dev.) (missing data, *n* = 10)	16.6 (1.22)	16.8 (1.05)	16.4 (1.28)	16.6 (1.34)
BMI 24 months Z-score: mean (Std. Dev.)	0.00 (1.00)	0.04 (0.55)	−0.05 (1.07)	0.07 (1.64)
Sex: n(%) (missing data, *n* = 0)				
Male	758 (48.6)	283 (48.0)	367 (48.5)	108 (50.9)
Female	800 (51.4)	306 (52.0)	390 (51.5)	104 (49.1)

*BMI* Body Mass Index, *IMD* Index of Multiple Deprivation

### Statistical analysis

Individual TV-time trajectories from 6 to 36 months of age were estimated using linear longitudinal multilevel models; with measurement visits at level one nested within children at level two. These models allow for the simultaneous measurement of within and between person differences, in conjunction with a number of time constant or time varying potential determinants. Time invariant (child level) and time variant (occasion level) measures can be incorporated into the model to assess their contribution to trajectories of child TV viewing. Multilevel models are able to accommodate data missing at random and unbalanced longitudinal designs, leading to more efficient estimates compared to methods that exclude cases to obtain balanced data [29]. The analysis strategy was to begin with a simple model, including only the age of the child to determine the average trajectory of TV-time. This model (model 1) contains polynomial terms for child age (age squared and age cubed) to allow for non-linear trajectories of child TV viewing. The second stage in the analysis then considers a range of covariates in isolation and then together in a multivariable model in order to determine whether socio-demographic, behavioural or attitudinal factors were associated with differences in the trajectories of child TV viewing. Time variant outcome and the determinants child age and mother TV-time are introduced into the model at the occasion level; all other determinants were time invariant or measured once and so were introduced into the model at the individual level. Results from these models are reported as model 2 and model 3 respectively. Covariates that are significant, and improve the model fit, are retained in the final model (model 4), and a number of interactions between significant covariates and child age were included. These interaction terms are used to determine whether the effect of socio-demographic, behavioural or attitudinal factors on child TV viewing change as the child becomes older. Analysis was carried out using Stata13. The predicted trajectories of child TV-time are given for particular groups of children, presented graphically in order to aid the interpretation of the model results. Trajectories of child TV-time are presented based on mother’s ethnicity and country of birth, and also for different scenarios based on mother’s attitude to TV viewing and the hours a TV is on in the house.

## Results

### Sample characteristics

Forty-nine per cent of mothers in the sample were of Pakistani ethnicity, of these 58% were born in Pakistan and 41% in the UK. Thirty-eight per cent of mothers were white British and nearly all were born in the UK. Table 1 describes the characteristics of the sample. Sixty-eight per cent of participants lived in the most materially deprived neighbourhoods of the UK (1st quintile in IMD 2010) and one third reported their financial situation to be difficult; only 1.5% lived in the least materially deprived neighbourhoods. On average, mothers were 27.5 ± 5.7 years old when their baby was born, and for 39% of mothers this was their first child. Just over a third of mothers were classified as experiencing psychological stress when their child was 12 months old. On average mothers were overweight (BMI = 25.9) and children (at 24 months old) had an average BMI of 16.6 (BMI z-score: 0.0 ± 1.0). Attrition in the study was minimal; however drop out was higher in mothers with lower than A-level education (72% of the sample at the beginning of the study and 59% by 36 months) and in mothers who reported having a difficult financial situation (41% of the sample at the beginning of the study and to 33% by 36 months).

### TV-time prevalence and trajectory

**Fig. 1:**
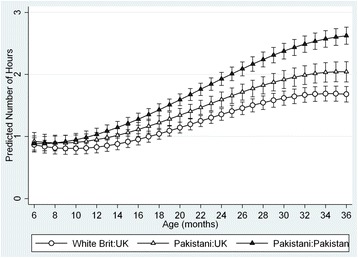
Estimated child TV-time by age, mother ethnicity, and mother’s country of birth

Model 1 (Table 2) represents the average trajectory of child TV-time, which is found to increase with age in a non-linear way. The estimated average trajectory is illustrated in Fig. 1.

**Table 2:** Summary model results

	Model 1	Model 2	Model 3	Model 4
	Estimate (95% C.I.)	Estimate (95% C.I.)	Estimate (95% C.I.)	Estimate (95% C.I.)
Constant	0.921 (0.848, 0.995)		−0.578 (−0.862, −0.294)	0.099 (−0.085, 0.283)
Fixed Effects				
Age	−0.026 (−0.048, −0.004)		−0.015 (−0.042, 0.012)	−0.054 (−0.081, −-0.027)
Age^2^	0.006 (0.004, 0.008)		0.006 (0.004, 0.008)	0.006 (0.004, −0.008)
Age^3^	−0.00012 (−0.00016, −0.00008)		−0.00011 (−0.00015, −0.00008)	−0.00011 (−0.00015, −0.00008)
Hours TV on in the home		0.067 (0.055, 0.079)	0.041 (0.027, 0.055)	0.020 (0.004, 0.036)
Mother TV-time		0.205 (0.187, 0.223)	0.192 (0.168, 0.216)	0.135 (0.104, 0.167)
Mother TV attitude ^a^		0.426 (0.314, 0.538)	0.249 (0.131, 0.367)	0.277 (0.167, 0.387)
Ethnicity (ref: White British)				
Pakistani		0.224 (0.129, 0.319)	0.219 (0.072, 0.366)	0.103 (−0.060, 0.266)
Other		0.155 (0.015, −0.295)	0.069 (−0.154, 0.292)	−0.007 (−0.262, 0.248)
Country Birth (ref: UK)				
Pakistan		0.247 (0.147, 0.346)	0.149 (0.008, 0.290)	−0.025 (−0.194, 0.144)
Other		0.148 (−0.006, 0.302)	0.170 (−0.083, 0.423)	0.137 (−0.153, 0.427)
In most deprived IMD quintile		0.166 (0.071, 0.260)	0.053 (−0.065, 0.171)	
No previous children		0.148 (0.056, 0.240)	0.072 (−0.042, 0.186)	0.098 (0.002, 0.194)
Child BMI at 24 months (z score)		−0.062 (−0.107, −0.018)	0.008 (−0.043, 0.059)	
Mother psychological stress (12 months) scored 4 or more.		0.06 (0.011, 0.109)	0.148 (0.042, 0.254)	0.133 (0.035, 0.231)
Interactions				
Age*Mother TV-time				0.004 (0.002, 0.006)
Age*TV on in the home				0.001 (0.001, 0.001)
Age*Pakistani ethnicity				0.012 (0.004, 0.020)
Age* Other ethnicity				0.005 (−0.009, 0.019)
Age*Pakistan born				0.014 (0.004, 0.024)
Age* Other non UK born				0.009 (−0.007, 0.026)
Random effects				
U_j(1)_ Random slope	0.385	0.254	0.254	0.228
U_j(2)_ Random intercept	0.001	0.0004	0.0004	0.0005
e_ij_ Between occasion	1.11	1.047	1.047	1.041
Total variance	1.496	1.3014	1.3014	1.269
Variance between children	26%	20%	20%	18%
Variance within children	74%	80%	80%	82%

Model 1: average trajectories of child TV viewing by age with a random effect for age along with age squared and age cubed to allow the modelling of non-linear trends

Model 2: reporting the coefficients from a series of univariate models considering each covariate independently

Model 3: as model 1 but with the inclusion of all covariates together in a multivariate model

Model 4: final model derived, as model 1 with all significant covariates and interaction terms in a multivariate model. This is the model used to estimate predicted child TV viewing

Variance between children is the percentage of variance in the outcome attributable to differences between children; variance within children is the percentage of variance attributable to change over time within children

All covariates are time invariant apart from mother TV time which was measured at each survey wave

*BMI* Body Mass Index, *IMD* Index of Multiple Deprivation

^a^Mother TV attitude: not agree that it is important that child not watch too much TV

On average, children’s estimated TV-time was less than one hour a day up to the age of 14 months 0.92 h (95% CI: 0.89, 0.95) (55 min) per day at 6 months old and 0.94 h (95%CI: 0.91, 0.97) (56 min per day at 12 months old). This was followed by a period of accelerated increase 1.28 h (95%CI: 1.25, 1.31) (77 min) per day at 18 months old, 1.71 h (95%CI: 1.67, 1.75) (103 min) per day at 24 months old), where TV-time rose to above two hours per day by 30 months (Fig. 1). Between 30 and 36 months, the rate of increase in TV-time was slower, increasing by approximately 15 min during this six-month period to 2.08 h (95%CI: 2.04, 2.12) (125 min) per day at 36 months old).

**Fig. 2:**
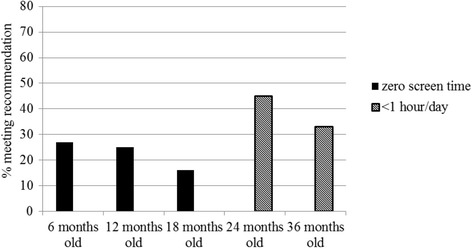
Percentage of children meeting international screen time recommendations

By 18 months old, it was estimated that only 16% of children met guidelines of zero screen viewing and by 36 months of age it was estimated that 33% met the guideline of <1 h screen viewing/day [11–13] (Fig. 2).

### Determinants of child TV-time

Mothers’ TV-time, the time the TV was on in the home, and mothers’ attitude towards child TV-time, all significantly predicted child’s TV-time when considered in univariate models (Table 2, model 2).

The effect sizes reduced when considered alongside other variables in multivariable models (Table 2, models 3), but they remain statistically significant. There was no association between how much mothers limited their child’s TV-time and the time the child spent in TV-time, this is therefore not reported in the models.

Model 4, which includes significant variables and interactions with age, predicted that there were already differences in child’s TV-time, associated with mothers’ TV-time, at the age of 6 months; and this association became stronger as the child grew older. For every extra hour that the mother watched TV, child TV-time was higher by 0.14 h (95%CI: 0.10–0.17) hours (8 min) at age 6 months and 0.26 h (95%CI: 0.30–0.37), (15 min) by age 36 months. For every hour the TV was on in the house children’s TV-time was 0.02 h (95%CI: 0.01–0.03) (1.2 min) higher at 6 months old, and 0.05 h (95%CI: 0.03–0.06) (3 min) higher at 36 months old.

Mothers’ attitudes to their child watching TV was associated with child TV-time. On average, children of mothers who did not agree that it was important that their child does not watch too much TV (23%), watched 0.28 h (95%CI: 0.17–0.39) hours (17 min) more TV-time daily compared to children of mothers who thought it was important that their child does not watch too much TV. There was no interaction between mothers’ attitudes and child age, and the effect was constant over the trajectory.

At 12 months old, children whose mothers’ had psychological stress had 0.13 h (95%CI:0.03–0.23), (8 min) more TV-time than children of mothers’ who did not have psychological stress (Table 2, model 4). There was no statistically significant association between mothers’ psychological stress and TV-time when children were 24 months old.

**Fig. 3:**
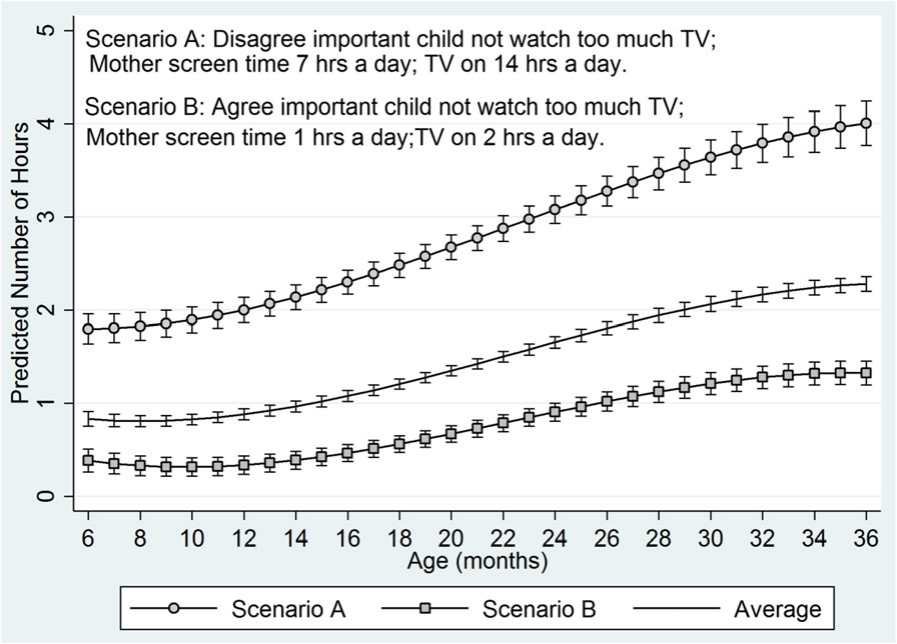
Average TV-time trajectory and combined effect of mother’s behaviour, attitude and stress on the trajectory

The modifiable factors that had a significant effect on the trajectories of child screen time were mother’s behaviour, attitude and stress. Figure 3 illustrates the combined effect of these variables by comparing two situations where mothers’ responses on all these variables are either not supportive (Scenario A, ‘worst case’ scenario) or supportive (Scenario B ‘best case scenario) of child’s TV-time. For comparative purposes the overall average trajectory of child TV-time is also shown in Fig. 3.

When ethnicity was considered in isolation in model 2, and with other covariates in model 3, it was found that the children of Pakistani ethnicity mothers had around 0.22 h (95%CI: 0.07–0.37) (13 min) more TV-time a day than children of white British mothers. The effect size remained similar in both models, such that ethnicity had an independent, separate effect after controlling for mothers’ behaviour and attitudes (and all other covariates).

There was a similar relationship between country of birth and child TV-time, although the effect size decreased more when considered in a multivariable model. Model 3 suggested that at 6 months of age, children of mothers born in Pakistan had around 0.15 (95%CI: 0.01–0.29) (9 min) more TV-time a day than children of mothers born in the UK.

In Model 4 there was an interaction between both ethnicity and country of birth and the age of the child. This is illustrated in Fig. 3. Once child TV-time began to increase, at around 12 months, the rate of increase was greater for children of Pakistani mothers born in Pakistan, than for children of white British and Pakistani mothers born in the UK. By the age of 36 months, children of Pakistani heritage mothers who were born in the UK had 0.4 h (22 min) extra TV-time compared to children of white British mothers; and children of Pakistani heritage mothers who were born in Pakistan, had 1.0 h (57 min) extra TV-time compared to children of white British mothers.

First-born children had more TV-time per day compared to those who were not (model 4, 0.1 ± 0.05 h; 6 min). A number of other variables were considered; these were not significant and so are not reported. These were mothers’ education, household tenure, mother’s BMI, child BMI, mother’s general health and child’s general health. Mother’s age at delivery was significant when considered alone in a univariate model; younger age being associated with increased child TV-time. However, once considered alongside other variables in Model 3, mother’s age was no longer significant (data not included in Table 2). Measures of self-reported financial situation and neighbourhood deprivation were significant when considered in isolation but not when considered in multivariable models (data not included in Table 2).

## Discussion

This prospective longitudinal study aimed to describe the prevalence, trajectory and determinants of TV-time in an ethnically diverse (predominantly white British and Pakistani) sample of children from the UK as they aged from 6- to 36-months-old. The study found that high levels of TV-time were common with an average estimated daily TV-time of 55 min at 6 months increasing to 124 min at 36 months. Modifiable determinants of young children’s screen time included mothers’ own TV-time, the time the television is on in the home and mothers’ attitude towards child TV-time. Mothers experiencing stress, first time mothers, and Pakistani heritage mothers, particularly those born outside of the UK, had children who had greater TV-time than their counterparts.

The high levels of TV-time reported in this study fall within the range reported in a systematic review of studies with children <2 years old from different countries (range: 36 to 197 min per day) [12]. In the same systematic review, [12] the proportion of children (aged <2 years old) meeting international recommendations (from Canada, Australia and the US [9–11]) of zero screen time was reported; results from the current study (27% at 6 months old, 16% at 18 months old) were similar to results from 9 out of 15 studies included in the review. By the age of 36 months, average TV-time of children in the current study was >2 h per day, which is higher than international guidelines (<1 h a day) [9–11]. Currently in the UK there are no specific guidelines for screen time, with guidance only stating that children under the age of five should minimise the amount of time spent being sedentary. Regardless, the amount of TV children in the study were watching is alarming given that the early years are critical for the development of health lifestyle behaviours [30]. The TV-time trajectory model showed that TV-time increased with age and a period of accelerated increase was observed between 12 and 30 months. This finding suggests that to maximise the effectiveness of early interventions, they should begin before the observed acceleration in TV-time at 12 months old.

In the current study the combined effect of modifiable parental influences (attitude, mother’s time spent watching TV, time TV on in the home) on child TV-time was predicted according to a ‘worst case’ scenario (scenario A) and a ‘best case’ scenario (scenario B). In the ‘worst case’ scenario children had almost 2 h of TV-time up to the age of 12 months old compared to less than half an hour a day in the ‘best case’ scenario. The rate of acceleration in TV-time after 12 months old was much greater in the ‘worst case’ compared to ‘best case’ and by age 36 months children in the ‘worst case’ had 4 h of TV-time a day (four times higher than international recommendations) compared to just over 1 h a day in the ‘best case’. Although children in the ‘best case’ scenario were still exceeding international recommendations the behaviour and attitudes that make up the ‘best case’ scenario could be viewed as useful and realistic targets to aim for in interventions to reduce TV-time. As reported in the findings, the only parental influence found not to be associated with the outcome of child TV-time was whether the mother reported restricting TV viewing of the child. There may be a number of reasons why this was not associated with the outcome, perhaps this measure is not accurately capturing maternal behaviour in this area.

Previous literature has reported inconsistent associations between young children’s screen viewing time and mother’s depressive symptoms [14]. In the present study we found that mothers psychological stress when the child was 12 months old significantly and independently predicted child TV-time, with around 8 min more viewing a day for those children of mothers with higher levels of psychological stress, but this was not the case when children were older (24 months). These findings may go some way towards explaining the variability in the findings of previous studies and underline the important finding that up to 12 months old is a key period for intervention. The findings suggest that mothers who are suffering from psychological stress postnatally should be a focus for intervention.

Previous studies have been inconsistent in their findings regarding the association between indicators of SES and screen viewing in early childhood [14]. This study found that, after controlling for other factors, there was no significant effect of neighbourhood deprivation or mothers’ self-reported financial situation upon TV-time when children are very young. However, older children and adolescents with a lower SES have consistently been reported to engage in higher levels of screen time [15–17] thus it is unclear when this socio-economic disparity begins; this requires further investigation.

Children from ethnic minority groups (Black and Hispanic) in the US have consistently been reported to have greater screen exposure in early childhood compared to their Caucasian counterparts [14]. The current study was the first to examine ethnic difference in young children’s TV-time outside of the US focusing on differences between white British and Pakistani heritage children in the UK. In the current study 49% of mother were of Pakistani heritage and 38% white British. This is broadly representative of the childhood population in Bradford where 47% of babies born are of South Asian heritage [22] and similar to other large UK cities where over one third of the population are of non-white ethnicity; therefore findings are relevant to these multi-ethnic populations. We found that children of mothers with Pakistani ethnicity overall had on average 13 min more daily screen time than their white British counterparts. The effect was independent of mothers’ behaviours and attitudes, and thus we cannot elucidate why these differences existed. Furthermore, we found an acculturation effect; children of mothers who were born in Pakistan had, on average, nine minutes greater TV-time at 6 months of age than children of mothers born in the UK. There was also an interaction between ethnicity, country of birth and the age of the child, such that the rate of increase in TV-time after 12 months of age was greater for children of Pakistani mothers born in Pakistan, than for children of mothers born in the UK. Our findings confirm those from the US and suggest that ethnic minority groups are particularly important targets for intervention. Qualitative explorations of TV-time behaviours in the different groups are required to begin to identify why differences exist between ethnic and cultural groups, and how behaviours can be modified.

### Implications for the development of effective interventions

To date few interventions to reduce screen time have targeted young children and those that have, have all focused on children aged 2 and above [31–34]. A systematic review of interventions reporting the results of 13 studies targeting children aged 2–5 years old found that those that were effective had greater parental involvement (usually in the form of parent education and training), however none explicitly targeted parents TV behaviours [35]. The current study found that mothers’ TV-time significantly and independently predicted their child’s TV-time, which replicates findings from a systematic review [14]. Further, this study found that the strength of this association increased as the children aged; by the age of 36 months every hour of mothers TV viewing was associated with an increase of around 25 min in child TV viewing. This suggests that mothers’ TV-time may be a key target for intervention. Furthermore, the strength of the association between mother and child TV-time increased as the children aged, thus supporting the idea that early intervention would reap greater effectiveness. The current study also found the time the TV was on in the house and mothers’ attitude towards their child’s TV-time were significant determinants of child TV-time. Figure 3 illustrates the size of the effect, and indicates how the size of this effect increases as the children get older; by the age of 36 months the differences in child TV-time can vary by over two hours a day. The strength of the association between time the TV was on and child TV-time also strengthened with age but this was not the case for mothers’ attitude towards their child’s TV viewing. A recent systematic review of interventions to reduce sedentary time in children and adolescents reported that encouraging a TV turn off week may be a promising strategy [36]. Since this would influence mother and child behaviours, testing the effectiveness of this for very young children would be worthwhile.

In a meta-analysis of interventions to reduce sedentary time in children and adolescents significant decreases in the amount of sedentary behaviour (post-intervention mean difference of −18 min/day) and BMI (post-intervention mean difference of −0.25 kg/m^2^) were found [35]. Thus, a reduction in sedentary time of 18 min/day could serve as a useful minimum threshold for interventions to aim for until sufficient evidence exist in younger children. In the current study, the effect size for each modifiable determinant alone was relatively small and unlikely to be clinically important in isolation. However, given their independence, changing these determinants in combination could lead to important changes in children’s TV-time. Therefore, evidence from the current study suggests that interventions to prevent excessive TV-time in children should include components that support mothers to: reduce their own TV-time, reduce the time the TV is on in the household and understand the importance of preventing excessive TV viewing during early childhood. One third of mothers in the current study reported experiencing stress, and this was associated with greater child TV-time. Parent programmes have been shown to reduce parental stress and depression [37], therefore interventions to reduce TV-time could include strategies from such programmes. Mothers experiencing stress, first time mothers, Pakistani ethnicity mothers, and mothers born outside the UK all had children with higher TV-time and thus should be particular targets for intervention; interventions should be appropriate and tailored for these groups.

### Strengths and limitations

The strengths of this study include its relatively large sample of children from a multiethnic, materially deprived population whose TV-time was measured at five time points over the first three years of life. The prospective longitudinal design enabled trajectories to be plotted and the identification of a potential key time point for intervention, 12 months of age, after which TV-time seemingly accelerates. It is the first study to report TV-time of young children from a South Asian ethnic minority group. Furthermore, the study examines acculturation in this ethnic minority group. The study is not without limitations, the questionnaire used to quantify child screen time has not been validated. It is a persisting problem that no valid or reliable tools are available for this age group [38]. The lack of variation in the neighbourhood material deprivation scores may have limited the ability to determine an association with the outcome. There was a systematic bias in missingness of data, mothers with lower educational attainment and those who reported having a difficult financial situation had higher rates of drop out. This may have affected the results; however, the number of participants in these groups who remained in the study was still high. The study only investigated the relationship between mother and child TV-time; the father/child relationship was not examined. Some literature suggests stronger relationships between sex matched parent/child dyads (mothers and daughters, fathers and sons), such that mothers have more influence on daughters and fathers on sons [39]. Whether this relationship exists when children are so young has not yet been investigated. The study did not explore whether mothers and children were watching TV together. Given the new American Academy of Pediatrics [11] recommendations that parents should co-view and re-teach screen content with their children, this would be interesting to investigate in future studies. We acknowledge that this study only measured TV-time and did not encompass other screen behaviours (e.g. tablet/computer use). However, in early childhood the main contributor to screen time is TV-time. [14] A further limitation is that screen viewing data was self/proxy reported and this brings with it recall limitations and possible social desirability bias, which may occur in the reporting of determinants (e.g. attitude) as well as TV-time. Moreover, we assumed no differential reporting error of TV-time between ethnic groups.

## Conclusion

High levels of TV-time were common amongst the children. A period of accelerated increase in TV-time was observed between 12 and 30 months, suggesting interventions should be targeted before this time. Modifiable determinants were identified and included mothers’ own TV-time, the time the television is on in the home and mothers’ attitude towards child TV-time. These behaviours may be key components to address in interventions for parents. Children of mothers experiencing stress, first time mothers, and mothers of Pakistani origin, particularly those born outside of the UK, had significantly greater TV-time than their counterparts and thus should be particular target groups for interventions to reduce TV-time during early childhood.

## Abbreviations

SES: Socioeconomic status
TV-time: Television viewing time

## References

[1] Caroli M, Argentieri L, Cardone M, Masi A (2004). Role of television in childhood obesity prevention. Int J Obes Relat Metab Disord.

[2] Marshall SJ, Biddle SJ, Gorley T, Cameron N, Murdy I (2004). Relationship between media use, body fatness and physical activity in children and youth: a meta-analysis. Int J Obes Relat Metab Disord.

[3] Rey-Lopez JP, Vicente-Rodriguez G, Biosca M, Moreno LA (2008). Sedentary behaviour and obesity development in children adolescents. Nurt Metab Cardiovas Dis.

[4] Jackson DM, Djafarian K, Stewart J, Speakman JR (2009). Increased television viewing is associated with elevated body fatness but not with lower total energy expenditure in children. Am J Clin Nutr.

[5] Ekelund U, Brarge S, Froberg K, Harro M, Aderssen SA, Sardinha LB, Riddoch C, Andersen LB (2006). TV viewing and physical activity are independently associated with metabolic risk in children: the European Youth Heart Study. PLoS Med.

[6] Hancox RJ, Milne BJ, Poulton R (2004). Associations between child and adolescent television viewing and adult health: a longitudinal birth cohort study. Lancet.

[7] Le Blanc AG, Spence JC, Carson V, Connor Gorber S, Dillman C, Janssen I, Kho ME, Stearns JA, Timmons BW, Tremblay MS (2012). Systematic review of sedentary behaviour and health indicators in the early years (aged 0–4 years). Appl Physiol Nutr Metab.

[8] Hinkley T, Verbestel V, Aherns W, Lissner L, Molnár D, Moreno LA (2014). Early childhood electronic media use as a predictor of poorer well-being: a prospective cohort study. JAMA Pediatr.

[9] Canadian Society for Exercise Physiology (2011). Canadian sedentary behavior guidelines.

[10] Australian Government Department of Health (2014). Move and play every day: national physical activity recommendations for children 0–5 years.

[11] AAP Council on Communications and Media (2016). Media and young minds. Pediatrics.

[12] Downing KL, Hnatiuk J, Hesketh KD (2015). Prevalence of sedentary behavior in children under 2 years: a systematic review. Prev Med.

[13] The NHS Information Centre for Health and Social Care: National child measurement Programme 2010/2011; http://content.digital.nhs.uk/catalogue/PUB03034/nati-chi-meas-prog-eng-2010-2011-rep1.pdf. Accessed 1 Aug 2016.

[14] Duch H, Fisher EM, Ensari I, Harrington A (2013). Screen time use in children under 3 years old: a systematic review of correlates. IJBNPA.

[15] Drenowatz C, Eisenmann JC, Pfeiffer KA, Welk G, Heelan K, Gentile D, Walsh D (2010). Influence of socio-economic status on habitual physical activity and sedentary behavior in 8-to 11-year old children. BMC Public Health.

[16] Brodersen NH, Steptoe A, Williamson S, Wardle J (2005). Sociodemographic, developmental, environmental, and psychological correlates of physical activity and sedentary behavior at age 11 to 12. Ann Behav Med.

[17] Fairclough SJ, Boddy LM, Hackett AF, Stratton G (2009). Associations between children’s socioeconomic status, weight status, and sex, with screen-based sedentary behaviours and sport participation. Int J Pediatr Obes.

[18] Biddle SJH, Pearson N, Ross GM, Braithwaite R. Tracking of sedentary behaviour of young people: a systematic review. Prev Med. 2010;51(5):345–51. 10.1016/j.ypmed.2010.07.018 20682330

[19] Jones RA, Hinkley T, Okelty AD, Salmon J (2013). Tracking physical activity and sedentary behaviour in childhood: a systematic review. Am J Prev Med.

[20] Tomopoulos S, Dreyer BP, Valdez P, Flynn V, Foley G, Berkule SB (2007). Media content and externalizing behaviors in Latino toddlers. Ambul Pediatr.

[21] Vandewater EA, Rideout VJ, Wartella EA, Huang X, Lee JH, Shim MS (2007). Digital childhood: electronic media and technology use among infants, toddlers and preschoolers. Pediatrics.

[22] Wright J, Small N, Raynor P, Tuffnell D, Bhopal R, Cameron N (2012). Cohort profile: the born in Bradford multi-ethnic family cohort study. Int J Epidemiol.

[23] Bryant M, Santorelli G, Fairley L, West J, Lawlor D, Bhopal R (2013). Design and characteristics of a new birth cohort to study the early origins and ethnic variation of child obesity: the BiB1000 study. Longitudinal Life Course Stud.

[24] Wareham NJ, Jakes RW, Rennie KL, Mitchell J, Hennings S, Day NE (2002). Validity and repeatability of the EPIC-Norfolk physical activity questionnaire. Int J Epidemiol.

[25] McMinn AM, van Sluijs EMF, Cooper C, Inskip HM, Godfry KM, Griffin SJ (2009). Validation of a maternal questionnaire on correlates of physical activity in pre-school children. IJBNPA.

[26] Office for National Statistics (2003). Ethnic group statistics: a guide for the collection and classification of ethnicity data.

[27] English Indices’ of Deprivation 2010: Overall. https://www.gov.uk/government/statistics/english-indices-of-deprivation-2010.

[28] Kessler RC, Andrews G, Colpe LJ (2002). Short screening scales to monitor population prevalence and trends in non-specific psychological distress. Psychol Med.

[29] Plewis I, Davis A, Davies RB (1994). Longitudinal multilevel models. Analyzing social and political change: a casebook of methods.

[30] Reilly JJ (2008). Physical activity, sedentary behaviour and energy balance in the preschool child: opportunities for early obesity prevention. Proc Nutr Soc.

[31] Schmidt ME, Haines J, O’Brian A, McDonald J, Price S, Sherry B (2012). Systematic review of effective strategies for reducing screen time among young children. Obesity (Silver Spring).

[32] Hinkley T, Cliff DP, Okely AD (2015). Reducing electronic media use in 2–3 year old children: feasibility and efficacy of the Family@play pilot randomised controlled trial. BMC Pub Heal.

[33] Birken CS, Maguire J, Mekky M, Manlhiot C, Beck CE, Degroot J (2012). Office-based randomized controlled trial to reduce screen time in preschool children. Pediatrics.

[34] Dennison BA, Russo TJ, Burdick PA, Jenkins PL (2004). An intervention to reduce television viewing by preschool children. Arch Pediatr Adolesc Med.

[35] van Grieken A, Ezendam NP, Paulis WD, van der Wouden JC, Raat H (2012). Primary prevention of overweight in children and adolescents: a meta-analysis of the effectiveness of interventions aiming to decrease sedentary behaviour. Int J Behav Nutr Phys Act.

[36] Altenburg TM, Kist-van Holthe J, Chinapaw MJ (2016). Effectiveness of intervention strategies exclusively targeting reductions in children’s sedentary time: a systematic review of the literature. Int J Behav Nutr Phys Act.

[37] Furlong M, McGilloway S, Bywater T, Hutchings J, Smith SM, Donnelly M. Behavioral and cognitive-behavioural group-based parenting interventions for early-onset conduct problems in children age 3–12 years. Cochrane Database Syst Rev. 2012; Issue 2. Art. No.: CD008225. 10.1002/14651858.CD008225.pub2 PMC12935172 22336837

[38] Hidding LM, Altenburg TM, Mokkink LB, Terwee CB, Chinapaw MJ. Systematic review of childhood sedentary behavior questionnaires: what do we know and what is next? Sports Med. 2016. 10.1007/s40279-016-0610-1 PMC5357243 27577686

[39] Abbott G, Hnatiuk J, Timperio A, Salmon J, Best K, Hesketh KD (2016). Cross sectional and longitudinal associations between parents and preschoolers physical activity and TV viewing: The HAPPY study. J Phys Act Health.

